# How Verbal Intelligence Predicts Emotional Stability: The Moderating Role of Empathy

**DOI:** 10.3390/jintelligence14060096

**Published:** 2026-06-01

**Authors:** Stanislava Stoyanova, Stoil Mavrodiev

**Affiliations:** Department of Psychology, South-West University “Neofit Rilski”, 2700 Blagoevgrad, Bulgaria; stoil_m@swu.bg

**Keywords:** 16PF, emotion regulation, emotional stability, empathy, moderating effect, verbal intelligence

## Abstract

This study examines whether verbal intelligence predicts emotional stability and investigates the moderating role of empathy in this relationship. Data were collected from 300 Bulgarian adults (*M* age = 29.6 years, *SD* = 12.8; 87.3% female) using Factor B (Reasoning/verbal crystallized intelligence) and Factor C (Emotional Stability) from Cattell’s 16PF Questionnaire (Form C), together with Yusupov’s empathy scale (Bulgarian adaptation). A general linear model (OLS regression) showed that higher verbal intelligence significantly predicted greater emotional stability (β = 0.116, *p* = .045, η^2^*p* = 0.013), while empathy had no significant main effect (*p* = .761). The interaction between verbal intelligence and empathy was significant (β = −0.138, *p* = .007, η^2^*p* = 0.024). Simple slopes analysis revealed that verbal intelligence positively predicted emotional stability at low (β = 0.253, *p* < .001) and medium levels of empathy (β = 0.116, *p* = .045), but the relationship became non-significant and slightly negative at high empathy levels (β = −0.022, *p* = .785). These findings suggest that verbal intelligence supports emotional stability most effectively when empathy is low to moderate, whereas high empathy may attenuate—or potentially reverse—this association, possibly due to increased empathic distress and reduced regulatory efficiency. The results underscore the importance of considering socio-emotional moderators when examining cognitive predictors of emotional adjustment and well-being.

## 1. Introduction

This paper examines how verbal intelligence predicts emotional stability, with empathy serving as a key moderating factor. Grounded in general (Spearman, 1904) and verbal and crystallized models of intelligence (R. B. Cattell, 1941), the study proposes that higher verbal intelligence supports stronger emotional stability through enhanced self-regulation, adaptive coping, and constructive positive self-talk. However, the strength and direction of this relationship depend on individuals’ empathic processing. Empathy may enhance this predictive relationship by facilitating social understanding and regulation of emotional responses. By exploring the interplay among verbal intelligence, emotional stability, and empathy, the study offers a more comprehensive understanding of how cognitive and socio-emotional capacities jointly contribute to psychological well-being and adaptive functioning.

Traditionally, intelligence and emotion were regarded as distinct domains until the emergence of emotional intelligence, conceptualized as the ability to perceive, understand, and regulate emotions (Mayer & Salovey, 1997). A central outcome of high emotional intelligence is emotional stability—characterized by sustained emotional balance, reduced susceptibility to negative affect, and quicker recovery from negative emotions (Chaturvedi & Chander, 2010). High emotional intelligence permits us to balance our emotions with the needs of those around us, thereby fostering harmonious relationships (Gergov & Andreeva, 2025). Research shows that individuals with high and low emotional intelligence perform similarly on neutral cognitive tasks (requiring attention), but individuals with high emotional intelligence outperform their peers on emotionally charged decision-making tasks (Checa & Fernández-Berrocal, 2019).

Both old and young adults rating higher their IQ also rate higher their emotional intelligence (Giannouli, 2023). Emotional intelligence has also been positively associated with verbal intelligence (Mayer et al., 2004, p. 210), suggesting a connection between cognitive–linguistic abilities and emotional processing. Moreover, verbal intelligence (a cognitive factor) and empathy (a social factor) both contribute to social adaptation (Carriedo et al., 2024). Effective psychosocial adaptation is typically marked by strong emotional, cognitive, and behavioral self-regulation; an internal locus of control; high self-esteem; lower stress; reduced affective empathy; greater perceived social and family support; reduced loneliness; and higher educational attainment (Carriedo et al., 2024). Together, these findings underscore the interconnected roles of cognitive and socio-emotional capacities in promoting psychological well-being and adaptive functioning through emotional regulation.

## 2. Emotional Stability

Emotional stability—conceptualized as low neuroticism—is one of the Big Five personality traits that predicts mental health, stress resilience, and overall well-being (Crnković et al., 2023; Oshio et al., 2018; Özer, 2022). Whereas neuroticism is characterized by frequent experiences of sadness, worry, and emotional volatility, high emotional stability reflects an individual’s tendency to experience calmness, resilience under stress, and psychological well-being (Crnković et al., 2023; Oshio et al., 2018; Özer, 2022). In this sense, emotional stability can be understood as an outcome of effective emotion regulation capacities.

Emotion regulation refers to the processes, such as cognitive control, through which individuals influence how they experience and express emotions (Paul, 2024). Cognitive control involves the regulation of thoughts, attention, and behavior in line with internal goals, drawing on executive functions such as working memory, inhibition, and task switching (Paul, 2024). Individuals with higher cognitive ability may possess stronger executive control over emotional responses, enabling more adaptive appraisal of stressors and more efficient affect regulation. Thus, cognitive control plays a central role in aligning emotional responses with long-term goals rather than short-term impulses (Paul, 2024).

## 3. Intelligence and Emotional Regulation

Intelligence, defined as the general cognitive ability to solve problems, reason, learn from experience (Colom et al., 2010), and achieve goals (Gignac & Szodorai, 2024), is a predictor of major life outcomes, including academic achievement (Roth et al., 2015), occupational performance (Deary et al., 2012; Schmidt & Hunter, 2004), and health status (Hemmingsson et al., 2006; Huh et al., 2024; Wrulich et al., 2014).

The association between intelligence and emotional stability is multifaceted. While higher emotional intelligence is linked to greater sensitivity to emotional information, individuals with higher IQs appear to process neutral and emotional information with comparable speed (Gillioz et al., 2023). Developmental findings further indicate that higher IQ is associated with psychological advantages: children and students with higher IQs report lower state and trait anxiety (Reynolds & Bradley, 1983; Scholwinski & Reynolds, 1985), and adolescents with higher fluid and crystallized intelligence demonstrate higher self-esteem and fewer conduct problems (Lavrijsen & Verschueren, 2023). Together, these findings highlight intelligence as a key cognitive resource linked not only to achievement but also to emotional and behavioral adjustment.

Some additional socio-emotional factors—such as empathy—may shape how intelligence relates to emotional functioning. Indeed, empathy has been identified as a mediator in the positive relationship between intelligence and prosocial behavior (Guo et al., 2023), operating through empathic prosocial motivational concern (Zaki & Ochsner, 2012). When intelligence is high, empathy can enhance emotional stability by fostering prosocial behavior and benevolence. Cognitive load reduces prosocial behavior, but eliciting empathy eases the impact of real-world cognitive load on prosocial behavior (Gamble et al., 2023).

## 4. Empathy and Emotional Regulation

Empathy—commonly divided into cognitive (understanding others’ emotions) and affective (experiencing someone else’s feelings) components (Davis, 1983; Decety & Jackson, 2004)—is a key socio-emotional capacity associated with interpersonal functioning and emotion regulation (Eisenberg et al., 2010; Preston & de Waal, 2002). Empathy enables individuals to accurately interpret others’ emotional states and respond appropriately.

Empathy comprises both a cognitive component—understanding another’s thoughts and emotions—and an affective component—sharing another’s emotional experience (Mu et al., 2025). These dimensions show distinct associations with emotional functioning. Greater cognitive empathy is linked to more effective emotion regulation, improving feelings, whereas greater affective empathy is associated with increased difficulties in regulating emotions (Mu et al., 2025; Thompson et al., 2021). Moreover, higher affective empathy for negative emotions is associated with poorer mental health outcomes, whereas higher affective empathy for positive emotions relates to better well-being (Larionow, 2025). The different dimensions of empathy may differentially shape emotional stability. The individuals with high cognitive empathy and high intelligence could be more emotionally stable than those with high intelligence and high affective empathy.

Empathy is a component of emotional intelligence (Mayer & Salovey, 1997; Mayer et al., 2004)—a construct encompassing the ability to identify, understand, and manage one’s own and others’ emotions (Fernández-Abascal & Martín-Díaz, 2019; Gómez-Leal et al., 2021). Individuals with higher emotional intelligence generally report greater empathic concern and stronger emotion regulation skills (Fernández-Abascal & Martín-Díaz, 2019; Gómez-Leal et al., 2021). Greater emotional understanding is linked to more intense evaluations of negative emotions, whereas higher emotion management is associated with a stronger emphasis on positive emotions (Fiori et al., 2024). The choice of interpersonal emotion regulation strategies (other-focused emotion regulation through social means) is influenced by interpersonal motivation to regulate emotions (Mu et al., 2025). Empathy contributes to emotional regulation processes by enhancing social awareness and perspective-taking. Empathy can either bolster stability through prosocial connection or undermine it via empathic distress, depending on the individual’s intellectual regulatory capacities.

Some research findings reveal that an increase in empathy is not accompanied by a change in emotional style encompassing intuition, situational sensitivity, resilience, outlook, self-awareness, and attention (Tsai, 2024), or even that there is a negative relationship between emotional stability and self-compassion (Özer, 2022). These findings suggest that the relationship of empathy with emotional stability is not necessarily linear.

## 5. Research Question

Does verbal intelligence predict emotional stability, and if so, how is this relationship moderated by empathy? Does the strength and/or direction of the relationship between verbal intelligence and emotional stability vary across different levels of empathy?

The moderation analysis was treated as exploratory given the limited prior evidence regarding the direction of this interaction.

## 6. Proposed Model

Moderation occurs when the strength or direction of a relationship between two variables depends on the level of a third variable that is the moderator (Baron & Kenny, 1986). In this framework, empathy functions as a moderator in the relationship between verbal intelligence and emotional stability, shaping how cognitive abilities translate into emotional adjustment. We propose that verbal intelligence predicts emotional stability, but that the strength of this prediction varies with empathy:

- Direct Path: Greater verbal intelligence leads to higher emotional stability. The reason for this could be better cognitive appraisal and the choice of appropriate emotion regulation strategies.
- Moderation by Empathy: Exploratory analyses will examine whether empathy amplifies the relationship between verbal intelligence and emotional stability by increasing sensitivity to both internal and external emotional cues, thereby promoting more deliberate and effective emotion regulation. Conversely, individuals with high verbal intelligence but lower empathy may rely more on rational, cognitively driven regulation strategies rather than emotionally attuned responses, which may strengthen the predictive effect of intelligence on emotional stability. Verbal intelligence may contribute most effectively to emotional stability when empathy is relatively low or moderate, as cognitive resources can be more directly translated into adaptive emotional regulation under conditions of lower affective reactivity. In contrast, high levels of empathy may attenuate this relationship, potentially due to greater susceptibility to empathic distress, emotional contagion, or reduced regulatory efficiency, which may interfere with the effective use of cognitive abilities in maintaining stable emotional functioning. Empathy may moderate the relationship between verbal intelligence and emotional stability such that the positive association is strongest at low to moderate levels of empathy and weaker at high levels of empathy, potentially due to increased empathic distress.

Intelligence allows an individual to process empathic information without becoming overwhelmed. In this context, intelligence predicts stability because the individual can maintain distinction between self and others, preventing the overidentification with the other person’s pain that leads to instability.

Conversely, empathy can moderate the relationship negatively if the individual lacks sufficient regulatory intelligence. In such cases, witnessing another’s suffering can trigger empathic distress, suggesting that suffering is perceived as a threat to the self, which may predispose to emotional instability. Thus, for individuals with high empathy but lower verbal intelligence, the ease of knowing what others are thinking may actually decrease emotional stability by increasing personal discomfort and worry.

## 7. Materials and Methods

This study was cross-sectional, conducted both online and offline through purposeful sampling of inviting only Bulgarians who were at least 18 years old to participate voluntarily in the study. Participation was voluntary. This study was conducted in accordance with the Declaration of Helsinki and approved by the Ethics Committee of the Independent Bulgarian Research Review Forum (protocol IBRRB02/5 February 2018).

### 7.1. Sample

The required sample size was *N* = 283, computed with Jamovi software 2.6.44 (Jamovi Project, 2025) for a general linear model with 3 degrees of freedom, for an effect size η^2^ = 0.03 with 1 degree of freedom and type I error rate set to 0.05, and this model was equivalent to a regression with 2 linear effect(s) and one 2-way interaction.

The participants in the study were 300 Bulgarians from 18 to 68 years old (*M* = 29.6, *SD* = 12.8)—38 men (12.7%) and 262 women (87.3%). About 21% of participants were only children, and 79% had siblings.

### 7.2. Instruments

Empathy was measured by a questionnaire created by Yusupov (1991), whose Bulgarian version was published by Alexandrova (2016), Dimchev et al. (2009), and Johnson (1999). It consists of 36 items, 18 of which measure empathy (Cronbach’s alpha = 0.735 in the current study), 8 items form a Lie scale (Cronbach’s alpha = 0.673 in the current study), and the other items are fillers that do not participate in computing test scores. The items that form part of the Empathy scale concern empathy for humans (parents, children, elderly, strangers, and people in general), animals, and characters from works of fiction (Kisova, 2017; Tiunova, 2017).

Intelligence/Reasoning and Emotional Stability were measured with two scales (B and C, correspondingly) from Cattell’s 16PF questionnaire form C with 105 items (R. B. Cattell, 1956), whose Bulgarian version was published by Stamenkova (2005), Batarshev (2008), and Valchinova (2017). The questionnaire is written at a fifth-grade reading level, and it is meant for use with people 16 years and older (H. E. P. Cattell & Mead, 2008). The items have a three-point answer format (H. E. P. Cattell & Mead, 2008). The scales represent trait dimensions, or continuums, which are bipolars, with a range of possible degrees for each trait measurable along the dimension (Ciccarelli & White, 2013).

The opposite poles of the scale ‘B’ are concrete reasoning, poorer judgement, incoherent thinking, and low mental capacity versus high mental capacity, the ability for abstract thinking, and fast learning (R. B. Cattell, 1945; H. B. Cattell, 1989; H. E. P. Cattell & Mead, 2008; Mantsha, 2002). The scale Intelligence/Reasoning measures general verbal crystallized intelligence (R. B. Cattell, 1945, 1956; Primi et al., 2014) as the ability to discern relationships in terms of how things stand relative to one another, recognizing analogies and similarities, and being able to classify events and form typologies (H. B. Cattell, 1989). Verbal/crystallized reasoning ability was assessed using 16PF Factor B, which serves as a brief proxy indicator rather than a comprehensive measure of verbal intelligence. Factor B consists of 8 items (R. B. Cattell, 1956) whose Cronbach’s alpha = 0.647 in the current study. Given its brief 8-item format and modest internal consistency, 16PF Factor B should be considered a proxy measure rather than a comprehensive assessment of verbal crystallized intelligence.

The scale Emotional Stability consists of 6 items whose Cronbach’s alpha = 0.698 in the current study. The opposite poles of the scale ‘C’ are emotional reactive instability (easily upset, worrying, and changeable) and weak ego versus emotional stability that is adaptive and mature and strong ego, being calm and unruffled (R. B. Cattell, 1945; H. B. Cattell, 1989; H. E. P. Cattell & Mead, 2008; Mantsha, 2002; Primi et al., 2014).

### 7.3. Data Analysis

Data were analyzed with Jamovi 2.6.44 applying general linear modeling (Jamovi Project, 2025) and with SPSS Version 31 (IBM Corp, 2025) computing descriptive statistics, Cronbach’s alpha, and Pearson correlations.

There were not any missing data. Data were analyzed using hierarchical ordinary least squares (OLS) regression to examine whether verbal/crystallized reasoning ability predicted emotional stability and whether this relationship was moderated by empathy.

Prior to analysis, predictor variables (16PF Factor B and empathy) were mean-centered to reduce potential multicollinearity and to facilitate interpretation of the interaction effect. The interaction term was then computed as the product of the centered predictor and moderator variables.

In Step 1, the main effects of verbal/crystallized reasoning ability and empathy were entered. In Step 2, the interaction term (Reasoning × Empathy) was added to test the moderation hypothesis.

OLS assumptions were examined prior to interpretation of the model, including normality of residuals (Kolmogorov–Smirnov = 0.057, *p* = .280); homoscedasticity (Breusch-Pagan test = 2.07, df = 3, *p* = .557, suggesting no evidence of heteroscedasticity); independence of errors (Durbin–Watson test = 1.95, *p* = 0.620, i.e., no evidence of autocorrelation in the residuals, supporting the assumption of independent errors); and multicollinearity (VIF values ranged from 1.02 for factor B score to 1.15 for the interaction between factor B and empathy, and tolerance values ranged from 0.873 for the interaction between factor B and empathy to 0.981 for factor B score, indicating no evidence of problematic multicollinearity among the predictors). Visual inspection of the residuals versus fitted values plot showed that residuals were randomly dispersed around zero without clear systematic curvature, supporting the assumption of linearity.

Cook’s distance was used to evaluate the influence of individual observations on the regression model. Values ranged from near zero to 0.218 (*M* = 0.003, *SD* = 0.013), with no cases exceeding conventional thresholds (Cook’s D > 1) (Cook & Weisberg, 1982), suggesting that the model estimates were not driven by any single influential observation.

For significant interaction effects, simple slopes analyses were conducted by estimating the association between reasoning ability and emotional stability at low (−1 SD), mean, and high (+1 SD) levels of empathy.

## 8. Results

Most participants did not indicate high social desirability in their answers (*N* = 295; 98.3%) on the Lie scale of the Empathy questionnaire. In our sample, 5 participants obtained elevated scores on this scale. However, these cases were retained in the analyses because social desirability could be regarded as a personality characteristic (as a higher-order factor for the five personality factors—extraversion, emotional stability, conscientiousness, agreeableness, and openness to experience, according to Bäckström, 2007) and because removing them did not meaningfully change the results.

The inclusion of descriptive statistics and zero-order correlations allows for initial inspection of bivariate relationships prior to multivariate modeling.

Table 1 presents the means, standard deviations, and zero-order correlations among the study variables. The mean scores indicate an average level of expression across the measured constructs. Emotional stability was significantly correlated only with verbal crystallized intelligence.

The General Linear Model examining the relationships between empathy, verbal intelligence, and their interaction on emotional stability used Ordinary Least Squares (OLS) and resulted in an *R*-squared value of 0.0431, indicating a low proportion of variance explained by the predictors.

The results in Table 2 indicate that empathy did not significantly predict emotional stability, but higher intelligence (as indexed by 16PF Factor B) predicted higher emotional stability. The interaction between empathy and intelligence was significant, suggesting that the effect of intelligence on emotional stability varied with empathy levels. The effect sizes for empathy were negligible, while intelligence and its interaction with empathy showed small effect sizes.

Simple effects of intelligence at different levels of empathy revealed significant results at the medium and low levels of empathy, but not at high levels of empathy (see Table 3).

Increasing empathy, emotional stability slightly diminished (see Table 4), but these differences were not statistically significant (see Table 2). Increasing verbal intelligence (reasoning), emotional stability increased (see Table 4), and these differences were statistically significant (see Table 2).

**Figure 1:**
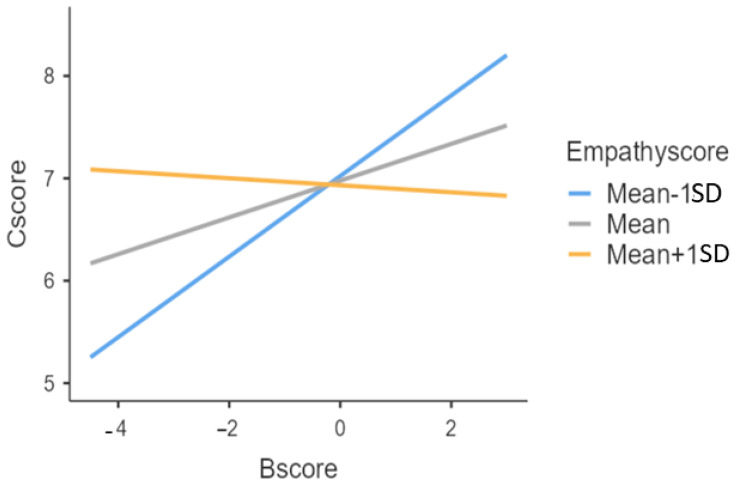
Change in emotional stability (Cscore) in dependence on intelligence (Bscore), empathy (Empathyscore), and their interaction.

Increasing verbal intelligence (as indexed by 16PF Factor B) at a low level of empathy increased emotional stability (see Table 2 and Table 5, and Figure 1). Increasing verbal intelligence at a medium level of empathy also increased emotional stability (see Table 2 and Table 5, and Figure 1). However, increasing verbal intelligence at a high level of empathy non-significantly diminished emotional stability (see Table 2 and Table 5, and Figure 1).

To examine robustness, sex (a dummy variable) was included as a covariate in the regression model. The pattern of results remained unchanged, with the interaction effect retaining significance. Including age as a covariate in the regression model also retained the significance of the interaction effect.

## 9. Discussion

The results of the present study indicate that verbal intelligence, as assessed by its proxy indicator Factor B of Cattell’s 16PF Questionnaire form C, significantly predicted higher emotional stability (β = 0.116, *p* = .045, η^2^*p* = 0.013), although the effect size was small. Stronger verbal reasoning abilities may support more effective cognitive appraisal of stressors, selection of adaptive coping strategies, and constructive positive self-talk, thereby contributing to greater emotional balance and resilience.

A significant interaction between verbal intelligence and empathy was observed (β = −0.138, *p* = .007, η^2^*p* = 0.024). Simple slopes analysis showed that verbal intelligence positively predicted emotional stability at low (β = 0.253, *p* < .001) and medium levels of empathy (β = 0.116, *p* = .045), but the relationship was non-significant and slightly negative at high levels of empathy (β = −0.022, *p* = .785). These results suggest that the positive association between verbal intelligence and emotional stability is most pronounced when empathy is low to moderate. At high levels of empathy, this association appears to be attenuated and may even become negligible or slightly reversed.

At low and medium levels of empathy, high intelligence provides the executive control needed to ensure that empathy leads to sympathetic concern rather than personal discomfort. However, a high level of empathy weakens the socio-emotional regulation capacities of high verbal intelligence. Intelligence predicts emotional stability most effectively when empathy is medium or low. While empathy connects us to others, it is the crucial component that enables transformation into either concern or distress.

One possible explanation is that high empathy, particularly its affective component, can lead to increased empathic distress or personal discomfort when individuals are confronted with others’ negative emotions. Such distress may reduce the regulatory efficiency otherwise afforded by verbal intelligence, potentially overwhelming cognitive resources needed for effective self-regulation and maintenance of self-other boundaries. In contrast, lower or moderate empathy may allow verbal intelligence to operate more effectively in the service of internal emotional regulation without the interference of excessive vicarious affect.

These findings highlight the importance of considering socio-emotional moderators when examining cognitive predictors of emotional adjustment. While verbal intelligence appears to function as a resource for emotional stability, the presence of high empathy may limit—or in some cases counteract—this protective effect. The results are in line with previous observations that affective empathy can be associated with greater difficulties in emotion regulation (Mu et al., 2025; Thompson et al., 2021) and poorer mental health outcomes under certain conditions (Larionow, 2025), whereas cognitive aspects of empathy may support more adaptive functioning (Mu et al., 2025; Thompson et al., 2021).

This model integrates cognitive and socio-emotional constructs in predicting emotional stability: verbal intelligence provides the cognitive toolkit for emotion regulation, whereas empathy enriches the emotional and social context in which these cognitive processes operate.

The overall proportion of variance explained by the model was modest (*R*^2^ = 0.043), indicating that verbal intelligence and empathy, together with their interaction, account for only a small part of individual differences in emotional stability. Other unmeasured variables, such as additional personality traits (e.g., openness to experience), specific emotion-regulation strategies, or contextual factors, likely play important roles.

Although the observed effect sizes were small, such findings may still be meaningful in the context of complex psychological systems, where behavior and personality traits are shaped by multiple interacting influences. Rather than representing strong or deterministic effects, the associations observed here may be better understood as context-dependent relational patterns that contribute incrementally to emotional stability. This perspective is consistent with applied statistical approaches in behavioral research, where modest but informative signals are often interpreted as context-dependent patterns within multifactorial processes rather than definitive evidence of underlying mechanisms (Zhao et al., 2025). The present findings suggest that verbal/crystallized reasoning ability and empathy are associated with emotional stability in a statistically significant but practically modest manner. These relationships should be interpreted as context-dependent patterns within a broader network of psychological influences rather than as evidence of direct or strong causal mechanisms.

### 9.1. Limitations

Empirical research directly testing the moderation of the intelligence–emotional stability link by empathy is limited. Several limitations should be noted. The sample was predominantly female (87.3%) and consisted of Bulgarian adults, which restricted the generalizability of the findings. Although supplementary analyses controlling for sex and age indicated similar patterns of results, future research should employ more balanced samples and explicitly model demographic influences to improve external validity.

The cross-sectional design precludes conclusions about directionality or causality. Furthermore, the empathy measure (Yusupov scale, Bulgarian adaptation) does not allow clear differentiation between cognitive and affective components of empathy, although the observed pattern is consistent with the potential costs of high affective empathy. Reliance on self-report instruments may also introduce common-method variance—the trend for relationships between variables to appear stronger or weaker when they are measured using the same assessment format (Baumgartner et al., 2021). To reduce common method variance, this study ensured anonymity (Cooper et al., 2020; Kock et al., 2021) and varied the number of possible answers per scale (Baumgartner & Weijters, 2021; Kock et al., 2021). The effect of social desirability was controlled using a Lie scale that indicated participants’ sincerity of answering.

A limitation of the present study concerns the operationalization of cognitive ability. Factor B of the 16PF is a brief indicator of reasoning ability and does not provide a comprehensive assessment of verbal intelligence or broader intellectual functioning. In addition, its internal consistency in the present sample was modest (α = 0.647), suggesting that findings should be interpreted with appropriate caution. Future research would benefit from using more extensive and psychometrically robust intelligence measures.

### 9.2. Future Research

Future research could benefit from longitudinal designs, larger and more diverse samples, and instruments that separately assess cognitive and affective empathy. Future studies may employ other standardized measures of intelligence, emotional stability, and empathy (distinguishing between its components) to empirically test the proposed moderation model in large, diverse samples. Furthermore, some other personality traits related to intelligence like openness to experience may contribute to emotional stability. Inclusion of additional moderators (e.g., mindfulness, reappraisal ability) and objective measures of emotion regulation would help clarify the mechanisms underlying the observed interaction.

## 10. Conclusions

While preliminary evidence supports the theoretical plausibility that intelligence predicts emotional stability, this relationship is likely nuanced and influenced by socio-emotional factors such as empathy. Understanding the moderating role of empathy deepens insight into the complex interplay of cognitive and emotional processes underlying psychological well-being.

Verbal/crystallized intelligence emerged as a significant, though modest, predictor of emotional stability by providing the tools for complex emotional regulation. However, this relationship is moderated by empathy. When high intelligence is paired with low or medium empathy, it produces a stable, benevolent individual capable of internal harmony. High empathy may instead predict lower stability due to the onset of empathic distress.

In conclusion, the present study provides preliminary evidence that verbal intelligence predicts emotional stability, but this relationship is moderated by empathy. The positive link is most evident at low to moderate levels of empathy, whereas high empathy may weaken or potentially reverse the association, possibly due to increased empathic distress. These results underscore the complex interplay between cognitive and socio-emotional factors in promoting psychological well-being and adaptive emotional functioning.

## Figures and Tables

**Table 1:** Descriptive statistics and correlations (*N* = 300).

Variable	*M*	*SD*	1	2	3
Emotional Stability	6.947	2.448	-	r = 0.135, *p* = .019	r = 0.048, *p* = .404
B score (Verbal Intelligence)	4.090	1.574	r = 0.135, *p* = .019	-	r = 0.092, *p* = .113
Empathy	53.187	10.417	r = 0.048, *p* = .404	r = 0.092, *p* = .113	-

**Table 2:** The results from the general linear model examining the relationships between empathy, verbal intelligence, and their interaction on emotional stability.

Variables	*SS*	*df*	*F*	*p*	η^2^*p*	Estimate	β
Intercept		296	2506	<.001	0.894	6.977 *	<0.001
Model	77.217	3	4.445	.004	0.043		
Empathy	0.536	1	0.093	0.761	<0.001	−0.004 **	−0.018
Intelligence	23.416	1	4.044	.045	0.013	0.180 ***	0.116
Empathy × Intelligence	42.245	1	7.296	.007	0.024	−0.021 ▪	−0.138
Residuals	1713.929	296					
Total	1791.147	299					

Note: confidence intervals * CI [6.703, 7.252]; ** CI [−0.032, 0.024]; *** CI [0.004, 0.355]; ▪ CI [−0.035, −0.006].

**Table 3:** Simple effects of intelligence on emotional stability.

Empathy Moderator Levels	*F*_(1,296)_	*p*	η^2^*p*	Estimate	β
Mean-1-SD	12.124	<.001	0.039	0.393 *	0.253
Mean	4.044	.045	0.013	0.180 **	0.116
Mean+1-SD	0.075	.785	<0.001	−0.034 ***	−0.022

Note: confidence intervals * CI [0.171, 0.616]; ** CI [0.004, 0.355]; *** CI [−0.281, 0.212].

**Table 4:** Estimated marginal means of emotional stability for empathy and intelligence as independent factors.

Variables	Mean-1SD	Mean	Mean+1SD
Empathy	7.02	6.98	6.93
Intelligence	6.69	6.98	7.26

**Table 5:** Estimated marginal means of emotional stability for empathy and intelligence as interacting factors.

Levels of Intelligence and Empathy	Mean
Low intelligence × Low empathy	6.40
Medium intelligence × Low empathy	7.02
High intelligence × Low empathy	7.46
Low intelligence × Medium empathy	6.69
Medium intelligence × Medium empathy	6.98
High intelligence × Medium empathy	7.26
Low intelligence × High empathy	6.99
Medium intelligence × High empathy	6.93
High intelligence × High empathy	6.88

## Data Availability

Data are available from the corresponding author upon a reasonable request.
